# Changes Over Time of Diffusion MRI in the White Matter of Aging Brain, a Good Predictor of Verbal Recall

**DOI:** 10.3389/fnagi.2020.00218

**Published:** 2020-08-14

**Authors:** Renaud Nicolas, Bassem Hiba, Bixente Dilharreguy, Elodie Barse, Marion Baillet, Manon Edde, Amandine Pelletier, Olivier Periot, Catherine Helmer, Michele Allard, Jean-François Dartigues, Hélène Amieva, Karine Pérès, Philippe Fernandez, Gwénaëlle Catheline

**Affiliations:** ^1^Université de Bordeaux, INCIA, UMR 5287—équipe NeuroImagerie et Cognition Humaine, Bordeaux, France; ^2^CNRS, INCIA, UMR 5287—équipe NeuroImagerie et Cognition Humaine, Bordeaux, France; ^3^Laboratoire Neuroimagerie et vie quotidienne, EPHE-PSL University, Bordeaux, France; ^4^Université de Bordeaux, ISPED, Centre INSERM U1219—Bordeaux Population Health Research Center, Bordeaux, France; ^5^INSERM, ISPED, Centre INSERM U1219—Bordeaux Population Heath Research Center, Bordeaux, France; ^6^Service de Médecine Nucléaire, CHU de Bordeaux, Bordeaux, France; ^7^CMRR, CHU de Bordeaux, Bordeaux, France

**Keywords:** verbal recall, white matter, cognitive aging predictors, gray matter atrophy, white matter hyperintensities (WMH), diffusion MRI imaging

## Abstract

**Objective**: Extensive research using water-diffusion MRI reported age-related modifications of cerebral White Matter (WM). Moreover, water-diffusion parameter modifications have been frequently associated with cognitive performances in the elderly sample, reinforcing the idea of aging inducing microstructural disconnection of the brain which in turn impacts cognition. However, only few studies really assessed over-time modifications of these parameters and their relationship with episodic memory outcome of elderly.

**Materials and Methods**: One-hundred and thirty elderly subjects without dementia (74.1 ± 4.1 years; 47% female) were included in this study. Diffusion tensor imaging (DTI) was performed at two-time points (3.49 ± 0.68 years apart), allowing the assessment of changes in water-diffusion parameters over time using a specific longitudinal pipeline. White matter hyperintensity (WMH) burden and gray matter (GM) atrophy were also measured on FLAIR and T1-weighted sequences collected during these two MRI sessions. Free and cued verbal recall scores assessed at the last follow-up of the cohort were used as episodic memory outcome. Changes in water-diffusion parameters over time were included in serial linear regression models to predict retrieval or storage ability of elderly.

**Results**: GM atrophy and an increase in mean diffusivity (MD) and WMH load between the two-time points were observed. The increase in MD was significantly correlated with WMH load and the different memory scores. In models accounting for the baseline cognitive score, GM atrophy, or WMH load, MD changes still significantly predict free verbal recall, and not total verbal recall, suggesting the specific association with the retrieval deficit in healthy aging.

**Conclusion**: In elderly, microstructural WM changes are good predictors of lower free verbal recall performances. Moreover, this contribution is not only driven by WMH load increase. This last observation is in line with studies reporting early water-diffusion modification in WM tissue during aging, resulting lately in the appearance of WMH on conventional MRI.

## Introduction

Age-related brain tissue degradations have been largely described through MRI over the last two decades. First, MRI studies were based on a single-point measurement of brain structure and demonstrated a significant association between this single-point measurement and chronological age (Bennett and Madden, 2014). More recent longitudinal imaging studies have confirmed this cross-sectional observation. For gray matter (GM), longitudinal studies demonstrated cortical volume reductions (Raz et al., 2010; Lemaitre et al., 2012), cortical thinning (Thambisetty et al., 2010; Fjell et al., 2014), and atrophy of deep GM structures (Dumurgier et al., 2012) and hippocampus (Persson et al., 2012). White matter (WM) modifications related to age have also been extensively described, firstly through the presence of White matter hyperintensity (WMH) observed on T2-weighted MRI (Hachinski et al., 1986; Zimmerman et al., 1986; de Leeuw et al., 2001; Srikanth et al., 2009). Although their pathogenesis is not fully understood, these WMH would reflect tissue alterations, including demyelination, gliosis, and axonal loss (Fazekas et al., 1993). Hypertension is one of the risk factors associated with (de Leeuw et al., 2002), and growing evidence suggests that WMH is related to cerebral small vessel disease (Wardlaw et al., 2013). More recently, diffusion MRI and more particularly diffusion tensor imaging (DTI) were used to describe the microstructural state of WM bundles. Here again, large effects of age on water-diffusion parameters have been described in several cross-sectional design studies (Salat et al., 2005; Charlton et al., 2006; Kennedy and Raz, 2009a, b) but also in some longitudinal design studies (Barrick et al., 2010; Sexton et al., 2014; Bender et al., 2016a,b). It appears that microstructural changes of WM, detected using dMRI, could occur before the appearance of WMH indicating a physiopathological continuum between these two processes (Maillard et al., 2013; Pelletier et al., 2017).

These DTI modifications of WM brain constitute one of the determinants of age-related cognitive decline (Brayne, 2007; Pantoni et al., 2007; Deary et al., 2009). Indeed, several cross-sectional studies have associated age-related modifications of brain tissues with various cognitive performances of the elderly including processing speed, executive function, and memory (see Salat et al., 2005; Bennett and Madden, 2014). In contrast, longitudinal analysis based on at least two MRI sessions is less reliable on this association. Some studies demonstrated an association with processing speed (Lövdén et al., 2014; Yang et al., 2015), fluid intelligence (Ritchie et al., 2015), executive function (Scott et al., 2017), and working memory (Charlton et al., 2010). Whether episodic memory is impacted by the changes in WM integrity over time has been very sparsely considered (Bender et al., 2016a). Since episodic memory decline is one of the hallmarks of Alzheimer’s disease (AD), the understanding of neural bases underlying episodic memory decline in aging is of crucial importance to elaborate efficient care of cognitive decline in elderly. Episodic memory performances are commonly assessed with verbal recall task in aging subjects, and its evaluation is a key point in the diagnosis of AD. The Free and Cued Selective Reminding test (FCSRT, Grober et al., 1988) is a verbal recall task that is recommended by the International Working Group for AD diagnostic criteria (Dubois et al., 2014). This test gives the opportunity to discriminate the retrieval deficit from the storage deficit using the analysis of its different subscores.

The aim of this study is to evaluate the predictive value of water-diffusion parameter changes, assessed using diffusion MRI, for verbal recall outcome in a healthy aging population. We probed WM integrity on two DTI scans, performed 3.49 ± 0.68 years apart. A specific longitudinal spatial processing based on the “full tensor registration” pipeline suggested by Keihaninejad et al. (2013) has been used in this study. This pipeline provides a specific within-subject registration, allowing an accurate measure of diffusion metrics changes occurring in WM over time.

## Materials and Methods

### Participants

In this study, the participants were a subset of the AMI (Agrica MSA IFR de Santé Publique, Aging Multidisciplinary Investigations) cohort, an epidemiological study conducted in residents of agricultural communities (Pérès et al., 2012). The AMI study started in 2007 and included 1,002 older individuals who were at least 65 years old at baseline and living in rural areas in South-West France. These individuals were randomly recruited from the databases of the Farmer Health Insurance System (MSA, Mutualité Sociale Agricole). The study was approved by the institutional human ethics review board, and all individuals in the sample provided written informed consent to participate.

Neuropsychological tests were administrated at home by a neuropsychologist at baseline (2007) and since every 2 years. A first MRI session was performed at the 2-year follow-up and a second one 4 years later (mean follow-up: 3.49 ± 0.68 years). Inclusion criteria required valid MRI data for the two sessions and one cognitive assessment within 2 years following the last MRI (mean interval: 1.33 ± 0.63 years). Eligible participants were free of dementia and free of major brain pathological disorders (tumor, stroke, severe WM pathologies). A total of 130 participants were included in the present study.

### Demographic, Cognitive, and Clinical Variables

Level of education was defined in three categories: no schooling, low educational level (primary school validated with a French certificate corresponding to 7 years of schooling), and high educational level (secondary school and above). Global cognitive functions were assessed using the MiniMental State Examination (MMSE; Folstein et al., 1975). Verbal recall was assessed with the Free and Cued Selective Reminding Test (FCSRT; Grober et al., 1988). The subject is asked to give back words of a previously learned list containing 16 words. Two scores were measured: the number of words recalled spontaneously (free recall) or after cueing (total recall) for three sessions of recall. Several clinical vascular factors were also considered here: body mass index (BMI, weight/height), the presence of hypertension defined as blood pressure ≥140/90 mm Hg, and self-reported diabetes. Participants with one or more copies of the ε4 allele were classified as APOE ε4 carriers.

### MRI Acquisition

MRI scans were obtained using an Achieva 3T scanner (Philips Medical System, Netherlands) with an 8-channel phased array head coil. Anatomical high-resolution T1-weighted images were acquired in transverse plan using a 3D Magnetization-Prepared Rapid Gradient-Echo (MPRAGE) pulse sequence with the following parameters: repetition time (TR) = 8.2 ms, echo time (TE) = 3.5 ms, 7° flip angle, field of view (FOV) = 256 × 256 mm^2^, 180 slices, no gap, and voxel size of 1 × 1 × 1 mm^3^. Fluid-attenuated inversion recovery (FLAIR) images were also acquired with the following parameters: TR = 11,000 ms, TE = 140 ms, inversion time (TI) = 2,800 ms, 90° flip angle, FOV = 230 × 172 mm^2^, 24 slices of 5 mm of thickness, and voxel size 0.72 × 1.20 × 5 mm^3^. Diffusion-weighted (DW) images were acquired using a spin-echo single-shot echo-planar imaging (EPI) pulse sequence with the following parameters: TR = 6,770 ms, TE = 60 ms, 90° flip angle, FOV = 224 × 224 mm^2^, 60 slices, no gap, and voxel size of 2 × 2 × 2 mm3, with *b*-value = 1,000 s/mm^2^ applied along 21 noncollinear diffusion encoding directions and one b0 image. To increase the signal-to-noise ratio (SNR), the DW acquisition was repeated in four successive runs; two runs were performed with a phase-encoding applied in the posterior–anterior (PA) direction and two runs with a phase-encoding applied in the anterior–posterior (AP) direction. These PA and AP DW images were used during the preprocessing for susceptibility artifact correction.

All acquisitions were aligned on the anterior commissure-posterior commissure plan.

### DTI Data Processing

A complete data quality control was performed on DW images, by checking distortions, artifacts, loss of SNR, signal, or anatomical abnormalities. DTI data were processed with FMRIB Software Library (FSL 5.0.2.^1^). The “topup” and “eddy” tools of FSL were then used to combine the two reversed phases of DW datasets into a single one corrected from susceptibilities, eddy currents, and motion effects (Andersson et al., 2016). For each subject, DW images were co-registered to the reference volume image (b0 image of the first PA dataset) with an affine transformation. DTI parametric maps, namely, fractional anisotropy (FA), mean diffusivity (MD), diffusion tensor eigenvalues (λ_1_, λ_2_, λ_3_), and diffusion eigenvectors (V_1_, V_2_, V_3_), were computed using the function “*dtifit*” in FMRIB’s Diffusion Toolbox. A visual quality control was done for each DTI map and for the accuracy of the sum-of-square residual maps.

#### Longitudinal Tensor-Based Registration

The scalar registrations of DTI maps, usually used with TBSS analyses (Keihaninejad et al., 2013; Bach et al., 2014), suffer from excessive smoothing, poor within-subject anatomical reliability, and poor mismatch of areas with high occurrence of anatomical variation (Keihaninejad et al., 2013; Tu et al., 2016; Vilgis et al., 2017). These drawbacks could compromise the capacity of longitudinal diffusion MRI studies to probe subtle microstructural changes of cerebral tissues.

In this study, the registration of full diffusion-tensor fields, implemented in DTI-TK software (Yushkevich et al., 2008) and available at http://dti-tk.sourceforge.net/pmwiki/pmwiki.php?n=Main.HomePage, has been used to register the two-time point diffusion MRI (dMRI) data-sets. This registration procedure has been shown to be the most accurate for within-subject registration of longitudinal images (Keihaninejad et al., 2013; Tu et al., 2016; Vilgis et al., 2017).

For spatial normalization, the DTI-TK’s optimized “aging template” from the IXI database^2^ has been used. This template is constructed from 51 normal elderly subjects (65–83 years old, 21 males and 30 females).

**Figure 1:**
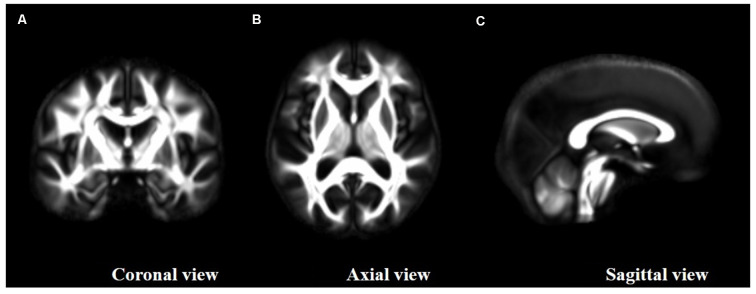
Study fractional anisotropy (FA)-template resulting from high signal-to-noise ratio (SNR) diffusion tensor imaging (DTI) maps and the group-wise registration of the full tensor ellipsoids as provided by DTI-TK. **(A)** Coronal view, **(B)** axial view and **(C)** sagital view.

Between-subject registration of the individual tensor ellipsoid procedure was applied on the preprocessed 131 × 2 dMRI datasets. Then, registered DTI parametric maps (FA, MD, λ_1_, λ_2_, λ_3_ maps) were computed from registered tensorial data for the first and second scans of each subject. A study-specific high SNR/quality FA template was obtained, averaging the 131 × 2 registered tensorial datasets (Figure 1 FA study template).

#### Longitudinal Tract-Based Spatial Statistics (TBSS)

The TBSS pipeline was applied to FA maps using a standard procedure (Keihaninejad et al., 2013; Bach et al., 2014). The study-specific FA template was thresholded at an FA value of 0.2 to prevent inclusion of GM voxels before creating a mean FA skeleton.

The longitudinal TBSS analyses were applied to all the other DTI metrics (FA, L1, L2, and L3). To simplify, the description of longitudinal TBSS analyses is only presented for the MD metric.

Skeletonized MD and ΔMD were then computed for all subjects, where each ΔMD represents the modification of the corresponding skeletonized MD between the first and second MRI scans.

Skeletonized ΔMD were computed voxel by voxel as follows:

$$\Delta \text{MD}=ΜD_{\text{MRI2}}-{\text{MD}}_{\text{MRI1}}$$

where MRI_1_ and MRI_2_ indices refer to the first and second MRI scans, respectively.

### MRI Biomarker Changes Over Time

#### White Matter MD Changes Over Time

A one-sample *t*-test was applied on the previously defined skeletonized ΔMD maps using the randomized program (Smith and Nichols, 2009) to generate t-statistic maps of MD increase. The t-statistic map corrected for multiple comparisons (*p* < 0.05, one-sample *t*-test, TFCE corrected, 5,000 permutations) was used to select the clusters presenting a real significant MD increase between the two time points. This thresholded *t-statistic* map was converted to a binary mask. This mask was then used to extract values from the ΔMD skeletonized maps to obtain quantitative values of WM diffusion parameter modification between the two MRIs for each subject.

#### Gray Matter Changes Over Time

A voxel-based morphometry (VBM) procedure implemented in the VBM8 Toolbox (revision 343^3^) of Statistical Parametric Mapping 8 (SPM8; Welcome Laboratory of the Department of Cognitive Neurology, Institute of Neurology, London, UK^4^) was used with default parameters to analyze brain volumes. Briefly, T1-weighted images were denoised, segmented into GM, WM, and cerebrospinal fluid (CSF) maps, warped to the Montreal Neurological Institute (MNI) space with a DARTEL-type nonlinear registration, and modulated to preserve volume information. An experienced operator visually checked all segmented maps to discard poor-quality processes.

Raw volumes of GM, WM, and CSF were extracted and expressed as the percentage of total intracranial volume (TIV), corresponding to the sum of GM, WM, and CSF volumes.

#### White Matter Hyperintensity (WMH) Load Change Over Time

WMH were obtained by the lesion growth algorithm (Schmidt et al., 2012) as implemented in the Lesion Segmentation Tool (LST) toolbox version 1.1.7^5^ for SPM8. Briefly, FLAIR images were co-registered to T1-weighted images using the intensity distribution of FLAIR images, outliers were detected, and lesion belief maps were calculated for each tissue classes (GM, WM, and CSF). These maps were then summed up, and a growth model lesion was applied to create lesion maps. Raw volumes of WMH were extracted from the generated maps.

### Statistical Analyses

According to an initial Shapiro–Wilk analysis, water-diffusion and WMH parameters do not present a normal distribution and for this reason these two MRI parameters were log-transformed before statistical analysis.

A first set of analysis was performed to explore the water-diffusion change over time in our sample. A regression model including age, sex, level of education, and ApoE4 status was built to investigate the demographic characteristics associated with the diffusion changes, followed by a matrix of correlation including the three imaging variables assessed here (Pearson’s correlation test).

In a second set of analysis, we investigate the association of water-diffusion metric changes with cognitive performances at the last follow-up. We first described the relationship of water-diffusion metric changes using either Pearson’s correlations (r) or Spearman’s (rho) correlation coefficients according to the distribution of the cognitive variables investigated. We then assessed the predictive value of water-diffusion metric changes over time for verbal recall performance measured at the last follow-up in a model accounting for the baseline verbal recall score; we have included baseline performance to account for the initial interindividual difference resulting from various demographic characteristics such as age at inclusion, sex, level of education, and ApoE4 status. Secondary models further adjusted for other MRI parameters (increased WMH load and loss of GM) or global cognitive level (MMSE) were built. To end, a regression model was built to investigate potential vascular risk factors associated with WM deterioration, including BMI, hypertension, and diabetes.

The results were considered as statistically significant when *p* < 0.05. All statistical analyses were performed using the IBM SPSS Statistics v. 22 software (IBM Corporation, Armonk, NY, USA).

## Results

The mean age of participants at the first MRI was 74.1 years (range = 66.6–85.5 years), and 47% were female (Table 1).

**Table 1:** Characteristics of participants (*n* = 130).

Variables	Mean ± SD or No. (%)
Demographic variables
Age at first MRI (years)	74.1 ± 4.7
Female	61 (47%)
Level of Education	
No schooling	42 (32%)
Low level	44 (34%)
High level	45 (34%)
APOE ε4 allele positive^1^	27 (21%)
Clinical variables of the last follow-up	
BMI (kg/m^2)^	26.4 ± 3.8
Hypertension^3^	55 (42%)
Diabetes^2^	18 (14%)
Cognitive variables of the last follow-up	
MMSE score^4^	26.7 ± 2.8
FCSRT free recall score^5^	22.9 ± 7.8
FCSRT total recall score^5^	42.8 ± 7.0
Brain tissue changes between the first and the second MRI	
Gray matter atrophy (cm^3^) (min-max)	16.79 ± 14.97 (−24.9–60.16)
Increase WMH lesion load (cm^3^) (min-max)	3.30 ± 4.86 (−0.9–34.11)
Increase white matter MD (μm^2^/s) (min-max)	53.26 ± 13.63 (29.23–98.68)

*Keys: SD, standard deviation; ^1^*n* = 106; ^2^*n* = 123; ^3^*n* = 123; ^4^*n* = 121; ^5^*n* = 109*.

### MRI Biomarkers Change Over Time

**Figure 2:**
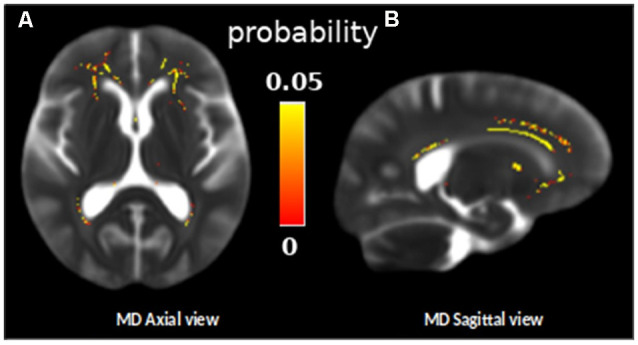
Skeleton ROIs in yellow presenting a significant increase of the mean diffusivity (MD) between the two diffusion MRI scans for the whole group, overlaid on a subject MD map (one sample *t*-test, *p* < 0.05, TFCE corrected). **(A)** Axial view and **(B)** sagital view.

The longitudinal TBSS analysis indicated a significant increase in MD in the frontal WM regions in our sample between the baseline and follow-up measurements (one-sample *t*-test, *p* < 0.05, TFCE corrected, Figure 2). The mean increase in MD in these frontal regions was 53.26 ± 13.63 μm^2^/s.

**Figure 3:**
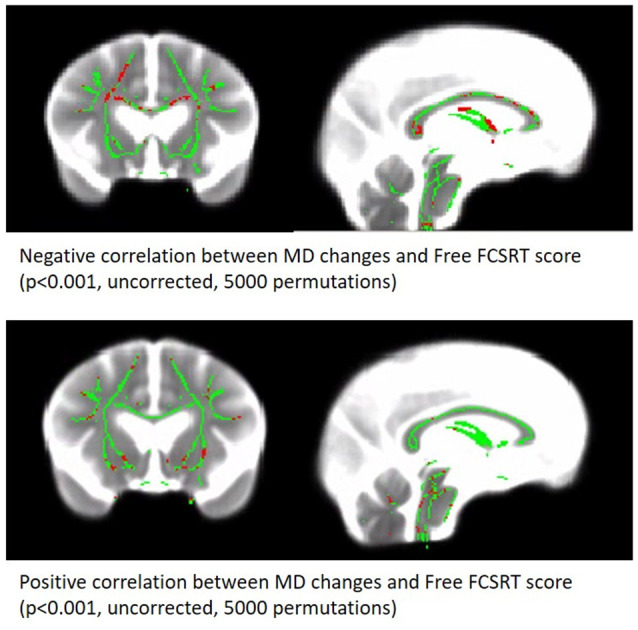
Correlation between MD changes Map and free verbal recall scores. Significant voxels are red (*p* < 0.001, uncorrected, 5,000 permutations). The results are presented on the skeleton of the cohort (green) display on a subject MD map.

As expected, subjects of the sample presented a decrease in GM volume (mean loss of 16.79 ± 14.97 cm^3^) and an increase in WMH load (mean increase of 3.30 ± 4.86 cm^3^) across the two MRI sessions (Table 1 and illustrated as spaghetti plots in Figure 3).

The first linear regression analysis revealed that the changes in WM MD over time were dependent on age at the first MRI (*t* = 3.49, *p* < 0.001) and not dependent on sex, level of education, or APOE ε4 status (Table 2).

**Table 2:** Linear regression model including WM-MD change over-time as a dependent variable and age, sex, level of education and ApoE4 status as explicative variables.

Predictor	Estimate	SE	*t*	*p*
Intercept	1.12866	0.17506	6.44738	<0.001
Age	0.00783	0.00224	3.49319	<0.001
SEXE	−0.00386	0.01945	−0.19824	0.843
Level of education	−1.08e−4	0.01256	−0.00862	0.993
APOE ε4 status	0.00232	0.02233	0.10411	0.917

*Model Fit R = 0.344, R^2^ = 0.118*.

The correlation matrix revealed significant associations between the WM-MD changes, the increased WMH load (*r* = 0.578, *p* < 0.001), and the GM atrophy (*r* = 0.172, *p* = 0.025; Table 3).

**Table 3:** Matrix of correlation between MRI biomarkers changes.

	ΔGM	ΔWMH	ΔMD
ΔGM	0.7	-	-
ΔWMH	0.195*^a^	12.5	-
ΔMD	0.172*^a^	0.578***^a^	1

Δ: difference between baseline and follow-up MRI data. ^a^Pearson’s correlation coefficient. *p < 0.05. ***p < 0.001.

### Prediction of Verbal Recall

Correlation analyses revealed that WM-MD changes are significantly associated with the MMSE score (rho = −0.169, *p* = 0.032), the free verbal recall (*r* = −0.289; *p* < 0.001), and the total verbal recall (rho = −0.182, *p* = 0.029). In the regression model accounting for the baseline performance, WM-MD change significantly predicts the score of the free recall of the last follow-up (*R*^2^ = 0.521, *β* = −0.207, *p* = 0.003). In contrast, WM-MD change does not predict neither the total recall of the last follow-up (*R*^2^ = 0.424, *β* = −0.116, *p* = 0.125) nor the MMSE (*R*^2^ = 0.459, *β* = −0.115, *p* = 0.099).

WM-MD change remained predictive of free verbal recall in secondary models adjusted either for increase in WMH burden (*R*^2^ = 0.524, *β* = −0.171, *p* = 0.042), GM atrophy (*R*^2^ = 0.529, *β* = −0.192, *p* = 0.007) or MMSE scores (*R*^2^ = 0.572, *β* = −0.164, *p* = 0.014).

Finally, the association between WM-MD change and free verbal recall is still significant in models adjusted for vascular risk factors (*R*^2^ = 0.532, *β* = −0.206, *p* = 0.004).

Supplemental analyses were performed on ΔFA and on Δλ1, Δλ2, and Δλ3 to disentangle the cellular process underlying the longitudinal changes of diffusion in our cohort. This analysis revealed that radial diffusion (Δλ2 and Δλ3) is a significant predictor of free verbal recall (*R*^2^ = 0.529, *β* = −0.227, *p* = 0.001 and *R*^2^ = 0.529, *β* = −0.226, *p* = 0.001 respectively for Δλ2 and Δλ3), whereas ΔFA and Δλ1 were not (see Supplementary Table S1).

## Discussion

In this study, we observed that WM MD increases in frontal regions predicted subsequent episodic memory performances in the elderly. This association was still significant in a model accounted for WMH load increase or GM atrophy measured over the same period, indicating that WM-MD changes in the frontal WM is the most predictive factor.

The specific changes of WM-MD in the frontal part of the brain observed here have already been described in previous longitudinal studies (Sexton et al., 2014) and associated with fluid intelligence (Ritchie et al., 2015), perceptual speed (Lövdén et al., 2014), reaction time (Yang et al., 2015), and working memory (Charlton et al., 2010). We reported here that verbal free recall scores are also predicted by these frontal WM changes. Considering GM, the FCSRT is usually associated with medial temporal regions (Sarazin et al., 2010; Pelletier et al., 2017). However, the ability to retrieve by your own the encoded items would indirectly involve attentional functions depending on the dorsolateral prefrontal cortex (Epelbaum et al., 2018). In accordance with this hypothesis, the MD changes observed here mainly concerned the rostrum, the anterior part of the corpus callosum. Fibers of the rostrum give rise to the forceps’ minor projection to extended frontal regions including the dorsolateral prefrontal cortex. Moreover, a previous study reports the contribution of the integrity of fibers coming from the anterior parts of the corpus callosum in cognitive control in aging (Strenziok et al., 2013). Another hypothesis would be a direct effect on the default mode network, the latter being highly implicated in the memory process. On the one hand, the posteromedial region which is a key structure of the network is highly connected with the medial temporal region and the modulation of the link between these two parts of the brain appears to be crucial in the encoding/retrieval process (Huijbers et al., 2012). On the other hand, the posteromedial region is also highly connected with the medial frontal region; this frontal part could be disconnected from the posterior part over time during aging, leading to a loss of efficiency in the network for memory task execution.

In addition to these frontal changes, we also observed MD changes over the fornix, which is the main output of the hippocampus. This last result is in agreement with several studies reporting age-related damage to the fornix (Pelletier et al., 2013; Kantarci, 2014; Metzler-Baddeley et al., 2019) and an association between diffusion properties of the fornix and episodic performances in the elderly (Metzler-Baddeley et al., 2011). More recently, a study showed that the microstructure of the fornix contributes to the episodic detail generation during retrieval (Memel et al., 2020).

Our results are clearly in accordance with the disconnection hypothesis for aging, proposed by Bennett and Madden (2014), since we observed a brain–behavior relationship based on within-person changes of water-diffusion metrics. Our result showing an effect of WM changes independently of GM modifications on cognitive outcome in the elderly is in accordance with a previous study (Hong et al., 2015). In a sample of 126 participants, WM changes were reported to be more related to processing speed before the age of 70, than to GM loss (Hong et al., 2015). They are also consistent with recent results showing an early alteration of the WM part of the limbic network in the healthy elderly (Gazes et al., 2019; Metzler-Baddeley et al., 2019). Interestingly, another recent study has shown that cognitive training could counteract the age-related WM degradation (de Lange et al., 2018).

The increase in MD observed here in the frontal WM area could rather reflect the demyelination process since we observed that λ2 and λ3 diffusion parameter changes were significantly associated with the verbal recall scores. In contrast, FA changes were not associated (data shown in Supplementary Material). The WM frontal regions included in the association analysis are a site of complex crossing fibers. We are aware that the tensorial model used in our study is not accurate to properly estimate FA in voxels containing a complex fiber architecture, which can explain the lack of association. According to previous studies, animal models of demyelination present a specific alteration of diffusion in the radial plane (Song et al., 2005; Tyszka et al., 2006; Wu et al., 2011), whereas animal models of axotomy present modification of diffusion in the axial plane (Song et al., 2003). However, the use of the diffusion metric pattern to interpret the underlying microstructural state of the tissue must be taken with caution since this association is a very complex phenomenon which depends on brain regions, pathological state of the tissue, and acquisition parameters used (Jones et al., 2013).

Extensive evidence indicates that several vascular factors, including hypertension, diabetes, and mid-life obesity, are associated with cognitive impairment and dementia in older adults (Duron and Hanon, 2008). More recently, a study performed on a large sample indicated that vascular burden and APOE ε4 status were associated with WM microstructural decline in cognitively normal older adults (Williams et al., 2019). All these factors could therefore account for a part of the association between WM water-diffusion parameters, assessed using MRI, and episodic memory observed in the present study. In our sample, the inclusion of vascular risk factors (overweight, hypertension, and diabetes) as covariates in the model did not affect the results.

Methodological limitations should be considered in data interpretation. The relationship between age-related water-diffusion changes in WM and amyloid deposition has been recently questioned (Song et al., 2018; Kim et al., 2019), and while conflicting results have been obtained (Rabin et al., 2018), we could not assess this aspect of the physiopathological process here since we do not have the amyloid status of our subjects. From a methodological point of view, we used TBSS statistical analysis, which only considers the skeleton of WM bundles. Moreover, our diffusion parameter estimation is based on only 21 directions. Future studies should be based on the use of multiple directions and multi-shell protocol to accurately assess free-water corrected diffusion parameters to disentangle extracellular modifications from cellular ones in aging brain tissue (Vipin et al., 2019).

In summary, we reported an association between change over time of the frontal WM-MD and verbal recall performances in older adults without dementia. The present study adds further evidence that alterations of structural connectivity in the elderly may constitute a risk factor for the development of episodic memory decline.

## Data Availability Statement

The datasets presented in this article are not readily available because institutional belonging. Requests to access the datasets should be directed to gwenaelle.catheline@u-bordeaux.fr.

## Ethics Statement

The studies involving human participants were reviewed and approved by the institutional human ethics review board of the Bordeaux CHU. The patients/participants provided their written informed consent to participate in this study. All procedures performed in studies involving human participants were in accordance with the ethical standards of the institutional and/or national research committee and with the 1964 Helsinki declaration and its later amendments or comparable ethical standards.

## Author Contributions

RN, BH, EB, BD, OP, AP, MA, PF, and GC: MRI acquisition and MRI processing. KP, HA, J-FD, and CH: set up and follow-up of the cohort. GC, MA, BH, ME, and MB: data analysis and manuscript writhing.

## Conflict of Interest

The authors declare that the research was conducted in the absence of any commercial or financial relationships that could be construed as a potential conflict of interest.

## Footnotes

^1^http://www.fmrib.ox.ac.uk/fsl

^2^https://brain-development.org/ixi-dataset/

^3^http://dbm.neuro.uni-jena.de/wordpress/vbm/download/

^4^http://www.fil.ion.ucl.ac.uk./spm

^5^www.applied-statistics.de/lst.html
