# Supplementary material for: Changes Over Time of Diffusion MRI in the White Matter of Aging Brain, a Good Predictor of Verbal Recall
Source: Front Aging Neurosci. 2020 Aug 14;12:218. doi: 10.3389/fnagi.2020.00218 (PMC7456903; doi:10.3389/fnagi.2020.00218)

Supplementary material

Other diffusion parameters

|  | | | | | | | | | | | |
| --- | --- | --- | --- | --- | --- | --- | --- | --- | --- | --- | --- |
|  |  |  |  |  |  |  |  |  |  |  |  |
| **Predictor** | | **Estimate** | | **SE** | | **t** | | **p** | | **stand β** | |
| Model Fit R=0.705, R^2^=0.497 | |  | |  | |  | |  | |  | |
| Intercept |  | 19.705 |  | 11.0135 |  | 1.79 |  | 0.076 |  |  |  |
| ∆λ1 |  | -10.832 |  | 5.6396 |  | -1.92 |  | 0.058 |  | -0.135 |  |
| Baseline FCSRT free recall score |  | 0.903 |  | 0.0941 |  | 9.60 |  | **< .001** |  | 0.674 |  |
| Model Fit R=0.727, R^2^=0.529 | |  | |  | |  | |  | |  | |
| Intercept |  | 38.545 |  | 12.0795 |  | 3.19 |  | 0.002 |  |  |  |
| ∆λ2 |  | -21.134 |  | 6.3430 |  | -3.33 |  | **0.001** |  | -0.227 |  |
| Baseline FCSRT free recall score |  | 0.880 |  | 0.0913 |  | 9.63 |  | **< .001** |  | 0.656 |  |
| Model Fit R=0.727, R^2^=0.529 | |  | |  | |  | |  | |  | |
| Intercept |  | 34.345 |  | 10.8573 |  | 3.16 |  | 0.002 |  |  |  |
| ∆λ3 |  | -19.687 |  | 5.9147 |  | -3.33 |  | **0.001** |  | -0.226 |  |
| Baseline FCSRT free recall score |  | 0.892 |  | 0.0908 |  | 9.83 |  | **< .001** |  | 0.666 |  |
| Model Fit R=0.692, R^2^=0.479 | |  | |  | |  | |  | |  | |
| Intercept |  | 1.933 |  | 13.8287 |  | 0.140 |  | 0.889 |  |  |  |
| ∆FA |  | -1.758 |  | 8.3942 |  | -0.209 |  | 0.835 |  | -0.0152 |  |
| Baseline FCSRT free recall score |  | 0.923 |  | 0.0970 |  | 9.510 |  | **< .001** |  | 0.6887 |  |

Table 1

MD results are mainly supported by radial diffusivity changes suggesting that demyelination could underly the diffusion estimate observed here.

Analysis of specificity

In order to test the specificity of the relationship described here between MD changes and verbal recall performances, we made additional analysis with various cognitive scores including the delayed verbal recall performances (free and total) and Weschler’s code score. The analyses on delayed FCSRT score are in agreement with the results observed with the FCSRT scores. MD changes are significantly associated with delayed free FCSRT (R^2^= 0.365, β= -0.175, p=0.029) score whereas it was not with the delayed total score. MD changes were also associated with the Weschler’s code score (R^2^= 0.685, β= -0.107, p=0.056).

These results confirm the specific association between the frontal MD changes and the retrieval ability estimated here through free scores. In contrast, the storage ability assessed through cued scores is not related to frontal MD changes. This retrieval ability has been classically associated with frontal brain networks. This hypothesis is consolidated by the observed association between MD changes and working memory score, a frontally mediated task.

|  | | | | | | | | | | | |
| --- | --- | --- | --- | --- | --- | --- | --- | --- | --- | --- | --- |
|  |  |  |  |  |  |  |  |  |  |  |  |
| **Predictor** | | **Estimate** | | **SE** | | **t** | | **p** | | **stand β** | |
| Model Fit R=0.705, R^2^=0.497 | |  | |  | |  | |  | |  | |
| Intercept |  | 19.705 |  | 11.0135 |  | 1.79 |  | 0.076 |  |  |  |
| ∆λ1 |  | -10.832 |  | 5.6396 |  | -1.92 |  | 0.058 |  | -0.135 |  |
| Baseline FCSRT free recall score |  | 0.903 |  | 0.0941 |  | 9.60 |  | **< .001** |  | 0.674 |  |
| Model Fit R=0.727, R^2^=0.529 | |  | |  | |  | |  | |  | |
| Intercept |  | 38.545 |  | 12.0795 |  | 3.19 |  | 0.002 |  |  |  |
| ∆λ2 |  | -21.134 |  | 6.3430 |  | -3.33 |  | **0.001** |  | -0.227 |  |
| Baseline FCSRT free recall score |  | 0.880 |  | 0.0913 |  | 9.63 |  | **< .001** |  | 0.656 |  |
| Model Fit R=0.727, R^2^=0.529 | |  | |  | |  | |  | |  | |
| Intercept |  | 34.345 |  | 10.8573 |  | 3.16 |  | 0.002 |  |  |  |
| ∆λ3 |  | -19.687 |  | 5.9147 |  | -3.33 |  | **0.001** |  | -0.226 |  |
| Baseline FCSRT free recall score |  | 0.892 |  | 0.0908 |  | 9.83 |  | **< .001** |  | 0.666 |  |
| Model Fit R=0.692, R^2^=0.479 | |  | |  | |  | |  | |  | |
| Intercept |  | 1.933 |  | 13.8287 |  | 0.140 |  | 0.889 |  |  |  |
| ∆FA |  | -1.758 |  | 8.3942 |  | -0.209 |  | 0.835 |  | -0.0152 |  |
| Baseline FCSRT free recall score |  | 0.923 |  | 0.0970 |  | 9.510 |  | **< .001** |  | 0.6887 |  |

Table 2


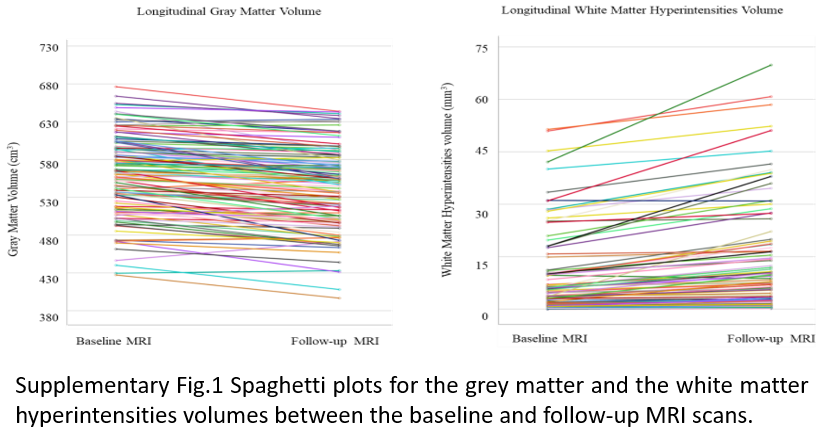

Supplement: Supplementary file 1 [file Data_Sheet_1.docx]
